# Supplementary material for: A system-wide network reconstruction of gene regulation and metabolism in Escherichia coli
Source: PLoS Comput Biol. 2019 May 3;15(5):e1006962. doi: 10.1371/journal.pcbi.1006962 (PMC6519848; doi:10.1371/journal.pcbi.1006962)
Supplement: S1 Text — Compilation of additional material for the integrative network model of E. coli. (PDF) [file pcbi.1006962.s001.pdf]

Supporting information for

A system-wide network reconstruction of gene regulation and metabolism in *Escherichia coli*

Anne Grimbs [1], David F. Klosik [2], Stefan Bornholdt [2], Marc-Thorsten Hütt [1*]

1 Computational Systems Biology, Department of Life Sciences & Chemistry, Jacobs University, Bremen, 28759, Germany
2 Institute for Theoretical Physics, University of Bremen, Bremen, 28359, Germany

* m.huett@jacobs-university.de

Table A. Vertex composition of the integrative *E. coli* network in total (Total) and for the partition regulatory domain – protein interface – metabolic domain (RD,PI,MD).

Vertices		Total	RD	PI	MD
⬢ (green hexagon)	*reaction*	4693	0	477	4216
■ (blue square)	*compound*	2681	0	26	2655
● (yellow circle)	*gene*	2545	1949	311	285
▲ (dark red triangle)	*protein monomer*	1917	198	1129	590
▲ (grey triangle)	*protein-protein complex*	929	65	243	621
▲ (purple triangle)	*protein-compound complex*	100	0	100	0
▲ (orange triangle)	*protein-rna complex*	3	1	0	2
		12868	2213	2286	8369

Table B. Edge composition of the integrative *E. coli* network in total (Total), for the partition regulatory domain – protein interface – metabolic domain (RD,PI,MD) and for the peripheral edges between the three domains (gray shading is only included to guide the eye).

Edges			Link	Total	MD	MD/PI	PI	PI/RD	RD	RD/MD
	gene - protein		D	1916	312	0	325	803	198	278
	protein - complex		C	1182	6	809	291	68	5	3
				4	0	0	4	0	0	0
				80	8	42	22	7	1	0
				6	0	0	0	6	0	0
	enzyme - reaction		D	1272	1225	5	42	0	0	0
				2775	2657	6	109	1	0	2
	educt - reaction		C	7707	7374	298	35	0	0	0
				246	0	1	245	0	0	0
				181	0	3	178	0	0	0
				100	0	0	100	0	0	0
	reaction - product		D	8303	7892	398	13	0	0	0
				171	0	1	170	0	0	0
				252	0	7	210	35	0	0
				102	0	0	102	0	0	0
	transport		C	291	281	8	2	0	0	0
	regulation	3,8	R	207	0	0	0	0	207	0
		1,2,9		1274	0	0	0	376	898	0
		12,14		9	4	1	2	1	1	0
		12		5	2	2	1	0	0	0
		13		4	0	2	2	0	0	0
		1,4,9		2082	0	0	0	185	1897	0
		12		2	0	1	1	0	0	0
		12		11	6	1	1	0	1	2
		13		1	0	1	0	0	0	0
		1,2		1160	0	0	0	1160	0	0
		9		2	0	0	0	0	2	0
		12		2	2	0	0	0	0	0
		2,5,6,7,10,11		98	0	0	0	0	0	98
		12		701	650	50	1	0	0	0
		12		1728	1667	59	0	0	0	2
		13		10	0	8	2	0	0	0
				31880	22086	1703	1854	2642	3210	385

C – Conjunct encoding; D – Disjunct encoding; R – Regulation

Table C. List of the 14 different kinds of regulatory processes subsumed in the edge type *regulation* of the integrative *E. coli* network (EcoCyc, release 20.0). Each of the 7296 regulatory processes comprises the regulator source ('Regulator') and target ('Regulated entity') as well as the regulatory mode, namely activation (+) and inhibition (−).

	Regulation type	#	Regulator	Regulated entity
1	Transcription-Factor-Binding	4302	Protein	Transunit, Promoter
2	Allosteric-Regulation-of-RNAP	219	Protein	Promoter
3	Ribosome-Mediated-Attenuation	12	RNA	Terminator
4	Protein-Mediated-Attenuation	5	Protein	Transunit, Terminator
5	Transcriptional-Attenuation	3	Compound	Transunit, Terminator
6	Rho-Blocking-Antitermination	3	Compound	Terminator
7	Small-Molecule-Mediated-Attenuation	2	Compound	Transunit, Terminator
8	RNA-Mediated-Translation-Regulation	195	RNA	Transunit, Gene
9	Protein-Mediated-Translation-Regulation	56	Protein	Transunit, Gene
10	Compound-Mediated-Translation-Regulation	22	Protein	Transunit, Gene
11	Regulation-of-Translation	4	Compound	Transunit, Gene
12	Regulation-of-Enzyme-Activity	2456	Compound, Protein	Enzyme
13	Regulation-of-Reactions	15	Compound, Protein	Reaction
14	Regulation	2	Protein	Protein

Table D. Comparison of vertex composition of the integrative *E. coli* network and the coverage to the integrative model from [1], the metabolic model from [2], and the transcriptional regulatory network based on the RegulonDB [3].

Vertices of the integrative *E. coli* network		*i*MC1010*	*i*AF1260**	RegulonDB
Reaction	4693	569/ 767	665/1436	0/ 0
Compound	2681	557/ 615	607/ 963	0/ 0
Gene	2545	971/1010	1168/1260	1764/1788
Protein monomer	1917			185/ 190
Protein-protein complex	929	771/ 817	0/ 0	11/ 13
Protein-compound complex	100			0/ 0
Protein-RNA complex	3			0/ 0
Total (model coverage)	12868	2868/3209	2440/3636	1960/1991
		89.4%	67.1%	98.4
Total (EcoCyc coverage)		3156/3209	3636/3636	1991/1991
		98.3%	100.0%	100.0%

* accounted only for intracellular reactions and unique metabolites, in total 1076 and 762

** accounted only for intracellular reactions and unique metabolites, in total 2382 and 1668

Table E. Most abundant vertices of the downwards (RD ⟶ MD) and upwards traversing paths (MD ⟶ RD), their quantity and the respective total degree and the corresponding degree rank.

Traversing paths		Vertex ID	Vertex name	Degree	Rank
RD	7304	PTSH-MONOMER	HPr (histidine protein)	37	74
↓	7276	PTSH-PHOSPHORYLATED	HPr-P (phosphorylated HPr)	35	79
MD	3642	RXN0-6718	EI-P + HPr → HPr-P + EI	4	4651
	2878				
	7304	PTSH-MONOMER	HPr (histidine protein)	37	74
	7276	PTSH-PHOSPHORYLATED	HPr-P (phosphorylated HPr)	35	79
	2496	RXN0-7166	PEP + HPr ↔ HPr-P + Pyr	4	4651
	2430				
	4464	RIBONUCLEOSIDE-DIP-REDUCTI-CPLX	RDPR1 (ribonucleoside-diphosphate reductase)	29	114
	2232	NRDA-MONOMER	RDPR1 subunit α	2	8434
	2232	B1-CPLX	RDPR1 α subunit dimer	2	8434
	2232				
	4464	RIBONUCLEOSIDE-DIP-REDUCTI-CPLX	RDPR1 (ribonucleoside-diphosphate reductase)	29	114
	2232	NRDB-MONOMER	RDPR1 subunit β	2	8434
	2232	B2-CPLX	RDPR1 β subunit dimer	2	8434
	2232				
	9324	of overall 18904			
MD	1822	PROTEIN-NRIP	NtrC-P (phosphorylated NtrC)	47	57
↓	1572	PROTEIN-NRIIP	NtrB-P (phosphorylated NtrB)	5	2879
RD	1349	NRIPHOS-RXN	NtrB-P + NtrC → NtrB + NtrC-P	4	4651
	1212	of overall 4070			
	**10536**	**of overall 22963**			

Table F. Compounds that serve as reactants of the phosphoenolpyruvate–carbohydrate phosphotransferase system, so-called PTS-sugars.

Vertex ID	Vertex name
N-ACETYL-D-GLUCOSAMINE_p	*N*-acetylglucosamine
N-ACETYL-D-MANNOSAMINE_p	*N*-acetylmannosamine
NACMUR_p	*N*-acetylmuramate
ASCORBATE_p	Ascorbate
CELLOBIOSE_p	Cellobiose
DIHYDROXYACETONE	Dihydroxyacetone
GALACTITOL_p	Galactitol
CPD-12538_p	Glucosamine
2-DEOXY-D-GLUCOSE_p	2-Deoxyglucose
CPD-15382_p	*keto*-Fructose
GLC_p	Glucose
CPD-3570_p	Methylglucoside
HYDROQUINONE-O-BETA-D-GLUCOPYRANOSIDE_p	Hydroquinone-O-glucopyranoside (arbutin)
MANNITOL_p	Mannitol
CPD-12601_p	Mannose
2-O-ALPHA-MANNOSYL-D-GLYCERATE_p	2-*O*-Mannosylglycerate
CPD-1142_p	Salicin
SORBITOL_p	Sorbitol
TREHALOSE_p	Trehalose

Table G. Regulated entities of the global transcriptional response regulator of the NtrBC system, the phosphorylated NtrC.

Vertex ID	Vertex name	Function of the encoded protein
EG10385	glnG	NtrC (inhibition)
EG10387	glnL	NtrB (inhibition)
EG10383	glnA	Glutamine synthetase (as 12-fold oligomer; inhibition)
EG12191	glnK	PII-2 (as trimer) can activate the adenylylation of glutamine synthetase
EG10386	glnH	glutamine ABC transporter - periplasmic binding protein
EG10388	glnP	glutamine ABC transporter - membrane subunit
EG10389	glnQ	glutamine ABC transporter - ATP binding subunit
EG11629	potF	putrescine ABC transporter - periplasmic binding protein
EG11630	potG	putrescine ABC transporter - ATP binding subunit
EG11631	potH	putrescine ABC transporter - membrane subunit
EG11632	potI	putrescine ABC transporter - membrane subunit
EG12124	hisJ	histidine ABC transporter - periplasmic binding protein
EG10007	hisM	arginine/histidine/lysine/ornithine ABC transporter - membrane subunit
EG10452	hisP	arginine/histidine/lysine/ornithine ABC transporter - ATP binding subunit
EG12125	hisQ	arginine/histidine/lysine/ornithine ABC transporter - membrane subunit
EG10072	argT	arginine/lysine/ornithine ABC transporter - periplasmic binding protein
EG11821	amtB	member of NH_3/NH_4^+ transporters, necessary for growth only at low NH_3 levels
G7071	cbl	Cbl DNA-binding transcriptional activator
G7072	nac	Nac DNA-binding transcriptional dual regulator
G6943	astA	Arginine succinyltransferase - 1st step in arginine degradation II (AST pathway)
G6941	astB	Succinylarginine dihydrolase (as dimer) - 2nd step in AST pathway
G6944	astC	Succinylornithine transaminase - 3rd step in AST pathway
G6942	astD	Succinylglutamate semialdehyde dehydrogenase - 4th step in AST pathway
G6940	astE	Succinylglutamate desuccinylase - 5th and final reaction in AST pathway
G6523	rutA	Uracil oxygenase - 1st step in uracil degradation III
G6522	rutB	peroxyureidoacrylate/ureidoacrylate amido hydrolase - 2nd step in uracil degradation III
G6521	rutC	(predicted aminoacrylate peracid reductase - 3rd step in uracil degradation III)
G6520	rutD	predicted aminoacrylate hydrolase - 4th step in uracil degradation III
G6519	rutE	predicted malonic semialdehyde reductase - 5th step in uracil degradation III
G6518	rutF	(flavin reductase - activity required for 1st step in uracil degradation (RutA))
G6517	rutG	member of the nucleobase:cation symporter-2 (NCS2) family of transporters (probably for uracil)
G6782	ddpX	D-Ala-D-Ala dipeptidase required for wild-type peptidoglycan biosynthesis
G6781	ddpA	(predicted peptide ABC transporter - periplasmic binding component)
G6780	ddpB	(predicted peptide ABC transporter - membrane component)
G6779	ddpC	(predicted peptide ABC transporter - membrane component)
G6778	ddpD	(predicted peptide ABC transporter - ATP-binding component)
G6777	ddpF	(predicted peptide ABC transporter - ATP-binding component)
G6969	yeaG	(impact in adaptation to sustained N starvation, member of Ser protein kinases)
G6970	yeaH	(impact in adaptation to sustained N starvation)
EG12834	yhdW	(predicted amino acid ABC transporter - membrane component)
EG12835	yhdX	(predicted amino acid ABC transporter - ATP-binding component)
EG12836	yhdY	(predicted amino acid ABC transporter - membrane component)
EG12837	yhdZ	(predicted amino acid ABC transporter - periplasmic binding component)

Table H. Hubs of the integrative *E. coli* network with a total degree of at least 50 (DC), their module affiliation, and the differentiation in in-degree (In) and out-degree (Out) including the affiliation and linkage assignments. The last column denotes the intra-domain degree fraction, ξ, here given as percental fraction.

			Affiliation				in-affiliation			in-linkage				out-affiliation			out-linkage			ξ [%]
DC	Vertex ID	Vertex name	RD	PI	MD	In	RD	PI	MD	C	D	R	Out	RD	PI	MD	C	D	R	
1412	`PROTON`	Proton			✓	1027		15	1012		1027		385	1	9	375	384		1	98.2
930	`WATER`	H_2O			✓	226		74	152		226		704		23	681	704			89.6
515	`CPLX0-226`	Crp-cAMP, transcriptional dual regulator		✓		1		1			1		514	513	1		1		513	0.4
489	`Pi`	Phosphate (P)			✓	410		8	402	1	409		79		2	77	40		39	98.0
439	`ATP`	ATP			✓	17		3	14		17		422		47	375	383		39	88.6
419	`PROTON_p`	Proton (periplasmic)			✓	96			96		96		323			323	323			100.0
401	`CPLX0-7534_o`	$[OmpF]_3$, outer membran porin F complex			✓	1		1		1			400			400		400		99.8
401	`CPLX0-7533_o`	$[OmpC]_3$, outer membran porin C complex			✓	1		1		1			400			400		400		99.8
399	`CPLX0-7530_o`	$[OmpE]_3$, outer membran porin E complex			✓	1		1		1			398			398		398		99.7
350	`ADP`	ADP			✓	299		35	264		299		51		9	42	22		29	87.4
293	`CPLX0-7797`	FNR, transcriptional dual regulator	✓			1	1			1			292	292					292	100.0
247	`WATER_p`	H_2O (periplasmic)			✓	21			21		21		226			226	226			100.0
241	`NAD`	NAD^+			✓	112		1	111	1	111		129		1	128	118		11	99.2
235	`NADH`	$NADH/H^+$			✓	115			115		115		120		1	119	110		10	99.6
222	`PC00027`	IHF, transcriptional dual regulator	✓			2		2		2			220	220					220	99.1
221	`CPLX0-7705`	Fis, transcriptional dual regulator	✓			1	1			1			220	220					220	100.0
187	`PD00288`	H-NS, transcriptional dual regulator	✓			1	1				1		186	186					186	100.0
178	`PPI`	Pyrophosphate			✓	147		8	139		147		31			31	9		22	95.5
177	`Pi_p`	Phosphate (periplasmic)			✓	168			168		168		9			9	3		6	100.0
176	`PHOSPHO-ARCA`	ArcA-P, transcriptional dual regulator	✓			3		3		1	2		173	173					173	98.3
159	`NADP`	$NADP^+$			✓	114		4	110		114		45		1	44	34		11	96.9
151	`NADPH`	$NADPH/H^+$			✓	33			33		33		118		5	113	114		4	96.7
144	`CO-A`	Coenzyme A			✓	50		2	48	1	49		94		6	88	77		17	94.4
138	`GLT`	Glutamate			✓	72			72		72		66			66	61		5	100.0
131	`CPLX0-7639`	$[Fur\text{-}Fe^{2+}]_2$, transcriptional dual regulator	✓			1		1		1			130	130					130	99.2
125	`2-KETOGLUTARATE`	2-Ketoglutarate			✓	58			58		58		67		2	65	59		8	98.4

Continued on next page

Continued

DC	Vertex ID	Vertex name	Affiliation RD	Affiliation PI	Affiliation MD	In	in-affiliation RD	in-affiliation PI	in-affiliation MD	in-linkage C	in-linkage D	in-linkage R	Out	out-affiliation RD	out-affiliation PI	out-affiliation MD	out-linkage C	out-linkage D	out-linkage R	ξ [%]
124	PHOSPHO-NARL	NarL-P, transcriptional dual regulator	✓			3		3		1	2		121	121					121	97.6
121	CPLX0-8070	DksA-ppGpp		✓		1		1			1		120	119	1		1		119	1.7
115	AMP	AMP			✓	80		8	72		80		35		1	34	10		25	92.2
111	PYRUVATE	Pyruvate			✓	59		5	54		59		52		5	47	36		16	91.0
98	AMMONIUM	NH_4^+			✓	70			70	1	69		28			28	13		15	100.0
87	CARBON-DIOXIDE	CO_2			✓	81		3	78		81		6			6	6			96.6
84	PC00061	Cra, transcriptional dual regulator		✓		3		3		1	2		81	79	2		2		79	6.0
82	CPLX0-3930	FlhDC, transcriptional dual regulator	✓			2		2		2			80	80					80	97.6
81	S-ADENOSYLMETHIONINE	Adenosylmethionine			✓	3		1	2		3		78	1	7	70	75		3	88.9
81	GUANOSINE_TETRAPHOSPHATE	Guanosine 5'-diphosphate 3'-diphosphate (ppGpp)			✓	4		1	3		4		77	64	1	12	2		75	18.5
78	CPLX0-8047	NsrR-nitric oxide		✓		1		1			1		77	76	1		1		76	2.6
76	ADENOSYL-HOMO-CYS	Adenosylhomocysteine			✓	65		4	61		65		11		3	8	1		10	90.8
71	ZN+2	Zinc (Zn^{2+})			✓	5		3	2		5		66		5	61	7		59	88.7
69	ACETYL-COA	acetyl-CoA			✓	33		1	32		33		36		3	33	28		8	94.2
68	OXYGEN-MOLECULE	O_2			✓	7			7		7		61		1	60	57		4	98.5
68	PHOSPHO-CPXR	CpxR, transcriptional dual regulator	✓			1		1		1			67	67			1		66	98.5
63	GTP	GTP			✓	2			2		2		61		2	59	41		20	96.8
63	MONOMER0-155	Lrp-Leucine, transcriptional dual regulator		✓		1		1			1		62	61	1		1		61	3.2
62	PHOSPHO-PHOB	PhoB-P, transcriptional dual regulator	✓			3		3		1	2		59	59					59	95.2
59	PC00010	LexA, transcriptional repressor	✓			1		1		1			58	58					58	98.3
58	PD00353	Lrp, transcriptional dual regulator		✓		2	1	1			2		56	55	1		1		55	3.4
58	CFA-CPLX	Cyclopropane fatty acyl phospholipid synthase			✓	9		1	8	1		8	49			49		49		98.3
57	PHOSPHO-PHOP	PhoP-P, transcriptional dual regulator	✓			2		2		1	1		55	55					55	96.5
54	AAS-MONOMER	Acyltransferase			✓	54			54		1	53								100.0
53	ALKAPHOSPHA-CPLX_p	Alkaline phosphatase (periplasmic)			✓	8		1	7	1		7	45			45		45		98.1
52	PHOSPHO-NARP	NarP-P, transcriptional dual regulator	✓			3		3		1	2		49	49					49	94.2
51	D-ALANINE	Alanine			✓	25		1	24		25		26		1	25	21		5	96.1
50	SUC	Sucrose			✓	23			23		23		27			27	22		5	100.0

Table I. Top betweenness central vertices of the integrative *E. coli* network and involved systems. The central reactions of each system are shaded in the corresponding system's color.

Betweenness	Vertex ID	Vertex name	GD	PI	MD	System (red)	(blue)	(yellow)	(teal)	(purple)	(black)	(green)	(steel blue)	(orange)	(olive)
0.29906	PROTON	Proton			✓	■		■				■	■	■	■
0.19894	WATER	H_2O			✓	■						■			
0.09670	ATP	ATP			✓	■	■	■					■	■	■
0.08320	Pi	Phosphate (P)			✓	■						■			
0.06466	PROTON_p	Proton (periplasmic)			✓	■									
0.06186	ADP	ADP			✓	■		■					■	■	■
0.05369	CAMP	cyclic-AMP (cAMP)			✓		■								
0.05362	RXN0-269_f			✓			■								
0.05362	CPLX0-226	Crp-cAMP, transcriptional dual regulator		✓			■								
0.05361	ADENYLATECYC-RXN				✓		■								
0.04082	PHOR-RXN			✓				■							
0.04066	PHOSPHO-PHOB	PhoB-P, transcriptional dual regulator	✓					■							
0.04056	PHOBR-RXN			✓				■							
0.04052	PHOSPHO-PHOR_i	PhoR, sensory histidine kinase (inner membrane)		✓				■							
0.03880	LEU	Leucine			✓				■						
0.03840	RXN0-261_f			✓					■						
0.03839	MONOMER0-155	Lrp-Leucine, transcriptional dual regulator		✓					■						
0.03722	FE+2	Ferrous (Fe^{2+})			✓					■					
0.03567	RXN0-5252_f			✓						■					
0.03567	CPLX0-7620	Fur-Fe^{2+}		✓						■					
0.03540	CPLX0-7639	[Fur-Fe^{2+}]$_2$, transcriptional dual regulator	✓							■					
0.03396	EG10671	ompF, outer membrane porin F gene	✓								■				
0.03395	EG10671-MONOMER	OmpF, outer membrane porin F		✓							■				
0.03395	CPLX0-7534_o	[OmpF]$_3$, outer membran porin F complex			✓						■				
0.03242	ADENYLATECYC-MONOMER	CyaA, adenylate cyclase			✓		■								
0.02743	GUANOSINE_TETRAPHOSPHATE	Guanosine 5'-diphosphate 3'-diphosphate (ppGpp)			✓							■			
0.02663	EG10670	ompC, outer membrane porin C gene	✓								■				
0.02663	EG10670-MONOMER	OmpC, outer membrane porin C		✓							■				
0.02663	CPLX0-7533_o	[OmpC]$_3$, outer membrane porin C complex			✓						■				

Continued on next page

Continued

Betweenness	Vertex ID	Vertex name	GD	PI	MD	System
0.02560	PPI	Pyrophosphate			✓	■
0.02489	WATER_p	H_2O (periplasmic)			✓	□
0.02432	EG10729	ompE, outer membrane porin E gene	✓			■
0.02432	MONOMER0-282	OmpE, outer membrane porin E		✓		■
0.02432	CPLX0-7530_o	$[OmpE]_3$, outer membran porin E complex			✓	■
0.02319	NAD	NAD^+			✓	□
0.02214	Pi_p	Phosphate (periplasmic)			✓	□
0.02119	PPPGPPHYDRO-RXN				✓	■
0.01890	ATPSYN-RXN_f				✓	■
0.01803	PYRUVATE	Pyruvate			✓	■
0.01786	GLT	Glutamate			✓	□
0.01607	CARBON-DIOXIDE	CO–2			✓	■
0.01603	NADP	$NADP^+$			✓	□
0.01353	PEPDEPHOS-RXN_2				✓	■
0.01252	PEPDEPHOS-RXN_1				✓	■
0.01173	ADENYLYLSULFKIN-RXN_r				✓	■
0.01171	AMMONIUM	NH_4^+			✓	■
0.01161	CARBAMATE-KINASE-RXN				✓	■
0.01144	CO-A	Coenzyme A			✓	□
0.01117	ABC-35-RXN_2				✓	■
0.01107	ABC-35-RXN_1				✓	■
0.01093	EG30063	micF, mRNA-interfering complementary RNA gene	✓			■

Continued on next page

Continued

Betweenness	Vertex ID	Vertex name	GD	PI	MD	System
	ATPSYN-RXN: ADP + Pi + 4 PROTON$_p$ ↔ ATP + WATER + 3 PROTON					
	ADENYLATECYC-RXN: ADENYLATECYC-MONOMER + ATP → CAMP + PPI; RXN0-269: PC00004 + CAMP → CPLX0-226					
	PHOR-RXN: PHOR-MONOMER$_i$ + ATP + Pi → PHOSPHO-PHOR$_i$ + ADP; PHOBR-RXN: PHOB-MONOMER + PHOSPHO-PHOR$_i$ → PHOSPHO-PHOB + PHOR-MONOMER$_i$					
	RXN0-261: PD00353 + LEU ↔ MONOMER0-155					
	RXN0-5252: PD00260 + FE+2 ↔ CPLX0-7620 ⇢ CPLX0-7639					
	EG30063 ⊣ EG10671 → EG10671-MONOMER ⇢ CPLX0-7534$_o$; EG10670 → EG10670-MONOMER ⇢ CPLX0-7533$_o$; EG10729 → MONOMER0-282 ⇢ CPLX0-7530$_o$					
	PPPGPPHYDRO-RXN: GDP-TP + WATER → GUANOSINE_TETRAPHOSPHATE + Pi + PROTON					
	PEPDEPHOS-RXN: PHOSPHO-ENOL-PYRUVATE + ADP + PROTON ↔ PYRUVATE + ATP					
	CARBAMATE-KINASE-RXN: CARBAMOYL-P + ADP + PROTON → CARBON-DIOXIDE + AMMONIUM + ATP					
	ADENYLYLSULFKIN-RXN: PAPS + ADP + PROTON ↔ APS + ATP					

Table J. Top ten key elements of the integrative *E. coli* network with respect to protein interface-specific degree (piDC) and betweenness centrality (piBC), respectively. The traversing path systems depict the embedding of system components in the protein interface (Figures 6 and 7 in the main text, Table E).

Rank	Vertex ID	Vertex name	piDC	piBC	Traverse path system
1	PTSH-MONOMER	HPr (histidine protein)	1	1	■
2	PTSH-PHOSPHORYLATED	HPr-P (phosphorylated HPr)	1	2	■
3	RED-THIOREDOXIN-MONOMER	$_{red}$Trx1 (reduced thioredoxin 1)	3	3	🟨
3	RED-THIOREDOXIN2-MONOMER	$_{red}$Trx2 (reduced thioredoxin 2)	3	3	🟨
5	OX-THIOREDOXIN-MONOMER	$_{ox}$Trx1 (oxidized thioredoxin 1)	3	5	🟨
5	OX-THIOREDOXIN2-MONOMER	$_{ox}$Trx2 (oxidized thioredoxin 2)	3	5	🟨
7	EG50003-MONOMER	acyl carrier protein (ACP)	8	14	□
8	FLAVODOXIN1-MONOMER	flavodoxin 1	10	17	□
9	OX-FLAVODOXIN1	oxidized flavodoxin 1	10	18	□
9	PROTEIN-CHEA	chemotaxis protein CheA	9	19	□
⋮					
12	RIBONUCLEOSIDE-DIP-REDUCTI-CPLX	RDPR1 (ribonucleoside-diphosphate reductase)	7	24	🟨
⋮					
20	RXN0-6718	EI-P + HPr → HPr-P + EI	63	7	■
⋮					
69	ADPREDUCT-RXN_f_1	NDP + $_{red}$Trx1 → dNDP + $_{ox}$Trx1 + H_2O	169	8	□
69	ADPREDUCT-RXN_f_2	NDP + $_{red}$Trx2 → dNDP + $_{ox}$Trx2 + H_2O	169	8	□
⋮					
72	THIOREDOXIN-REDUCT-NADPH-RXN_1	$_{ox}$Trx1 + NADPH/H^+ → $_{red}$Trx1 + $NADP^+$	169	9	□
72	THIOREDOXIN-REDUCT-NADPH-RXN_2	$_{ox}$Trx2 + NADPH/H^+ → $_{red}$Trx2 + $NADP^+$	169	9	□

■ RXN0-6718: PTSH-MONOMER + PTSI-PHOSPHORYLATED → PTSH-PHOSPHORYLATED + PTSI-MONOMER
🟨 RIBONUCLEOSIDE-DIP-REDUCTI-CPLX ⇢ RIBONUCLEOSIDE-DIP-REDUCTI-RXN:

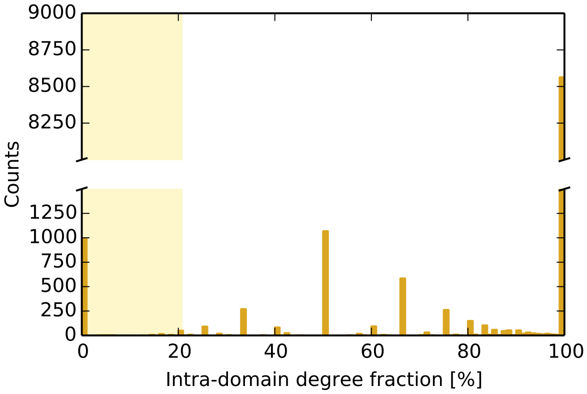

Fig A. Distribution of intra-domain degree fraction of the integrative *E. coli* network. The yellow shaded area represents the significant low intra-domain degree fractions tested via z-score.

Table K. Hubs and non-hubs of the integrative *E. coli* network with a significant low intra-domain degree fraction (ξ, tested via z-score) and a total degree (DC) larger than 12, and their domain affiliation (RD, PI and MD).

	Vertex name	RD	PI	MD	ξ	p-value	DC
Hubs	CRP-cAMP DNA-binding transcriptional dual regulator		✓		0.39	0.0042	515
	DksA-ppGpp		✓		1.65	0.0049	121
	Cra, transcriptional dual regulator		✓		5.95	0.0071	84
	Guanosine 5'-diphosphate 3'-diphosphate (ppGpp)			✓	18.52	0.0208	81
	NsrR-nitric oxide		✓		2.56	0.0052	78
	Lrp-Leucine, transcriptional dual regulator		✓		3.17	0.0055	63
	Lrp, transcriptional dual regulator		✓		3.45	0.0056	58
Non-hubs	ModE-MoO_4^{2-} DNA-binding transcriptional dual regulator		✓		4.17	0.006	48
	NtrC-P, transcriptional dual regulator		✓		8.51	0.0089	47
	NagC, transcriptional dual regulator		✓		4.35	0.0061	46
	PdhR, transcriptional dual regulator		✓		4.55	0.0062	44
	ArgR-arginine, transcriptional dual regulator		✓		5	0.0065	40
	DksA RNA polymerase-binding transcription factor		✓		5.13	0.0068	39
	PurR-Hypoxanthine, transcriptional repressor		✓		6.06	0.0071	33
	CysB-acetylserine, transcriptional dual regulator		✓		6.25	0.0072	32
	FhlA-Formate, transcriptional activator		✓		6.25	0.0072	32
	FadR, transcriptional dual regulator		✓		20.69	0.0245	29
	RutR, transcriptional dual regulator		✓		18.18	0.0202	22
	NsrR, transcriptional repressor		✓		14.29	0.0148	21
	CytR, transcriptional repressor		✓		18.75	0.0207	16
	AraC-arabinose, transcriptional activator		✓		12.5	0.0125	16
	GntR, transcriptional repressor		✓		20	0.0228	15
	DnaA-ATP, transcriptional dual regulator		✓		14.29	0.0148	14
	TrpR-Tryptophan, transcriptional repressor		✓		14.29	0.0148	14
	Mlc, transcriptional repressor		✓		15.38	0.0162	13

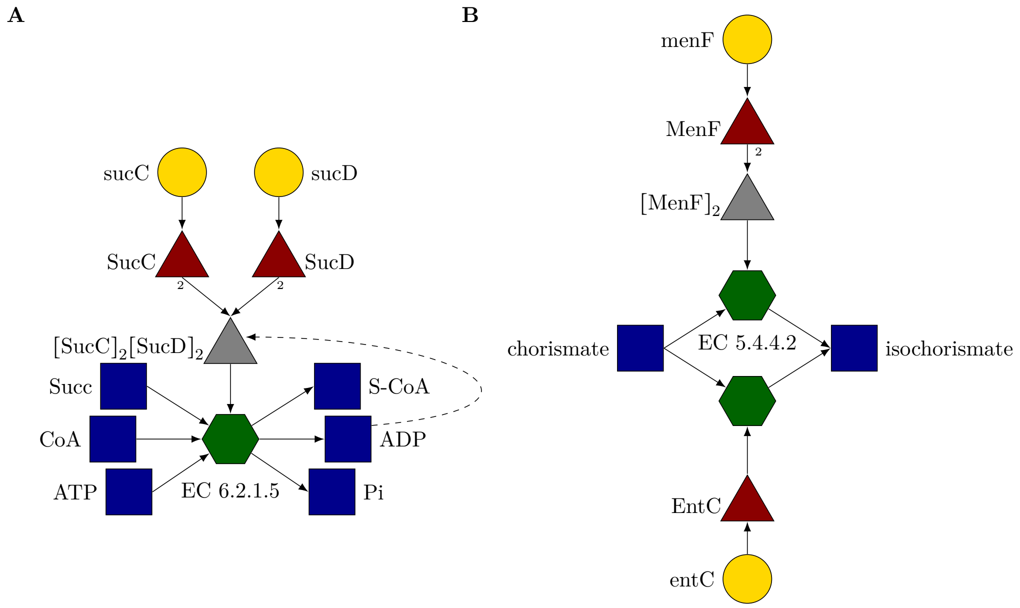

Fig B. Two biological examples of potential linkages. **A** succinyl-CoA synthetase (EC 6.2.1.5) and **B** isochorismate synthase (EC 5.4.4.2). The vertices are denoted by the common biological abbreviation (see EcoCyc webpage) and the respective biological category of a vertex (BCV): gene, protein monomer, protein-protein-complex, reaction, compound. The symbols are also explained in Figure 2 in the main text. The additional numbers indicate stoichiometric coefficients for the complex formation. This Figure has been adapted from the Supplementary Information of [4].

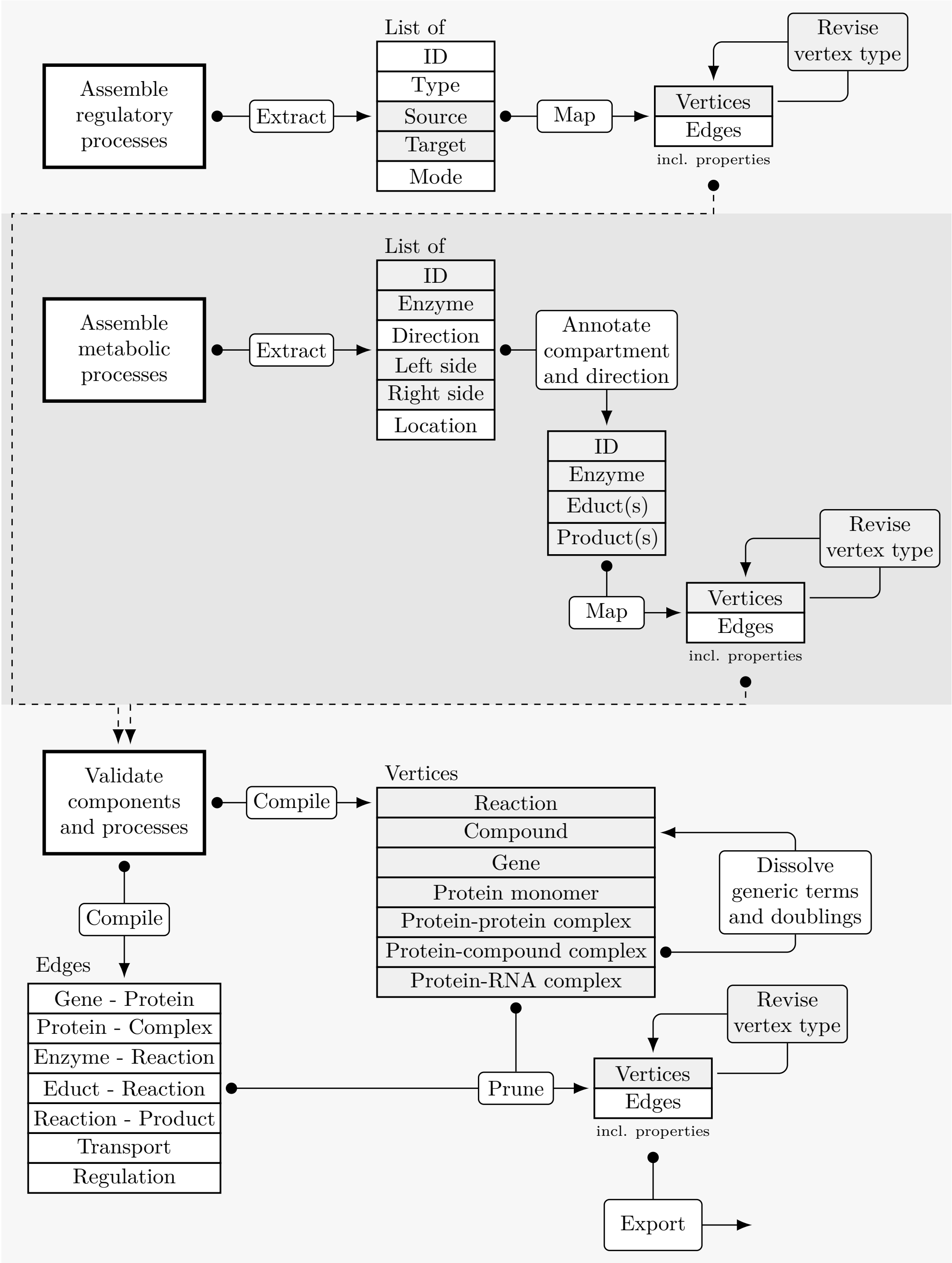

Fig C. Workflow of the integrative *E. coli* network reconstruction. The three major stages are marked by the thick boxes and are additionally emphasized by different gray-colored background. Following the arrows the process sequence can be traced including the operations (curved boxes) as well as the intermediate stages (corned boxes). The dashed lines describe the merging step of the first two stages to the third and final stage. The bulleted end of an arrow depicts the information usage of the entire box compilation, while an ordinary arrow ending means that only the information of the tagged individual box is used. The shading of a box denotes that the respective information is used for the definition of the graph vertices. A detailed description as pseudo code is given in Algorithm 1 in the main text.

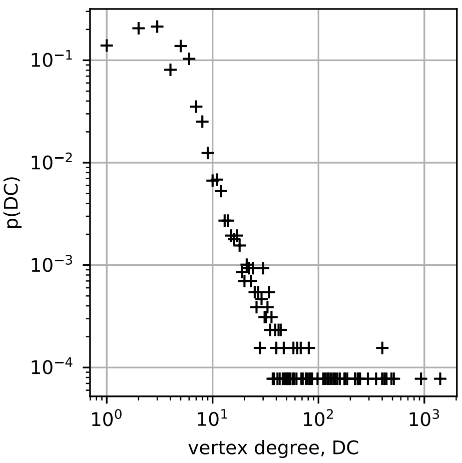

Fig D. Distribution of the total degree of the network reconstruction.

(a)

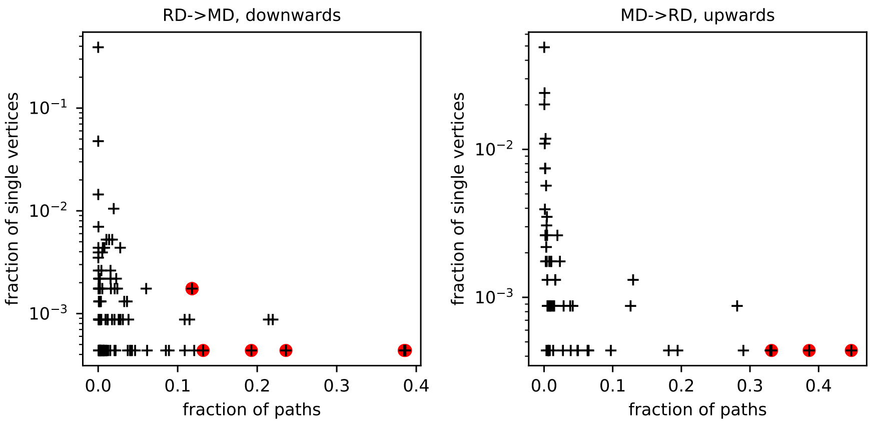

(b)

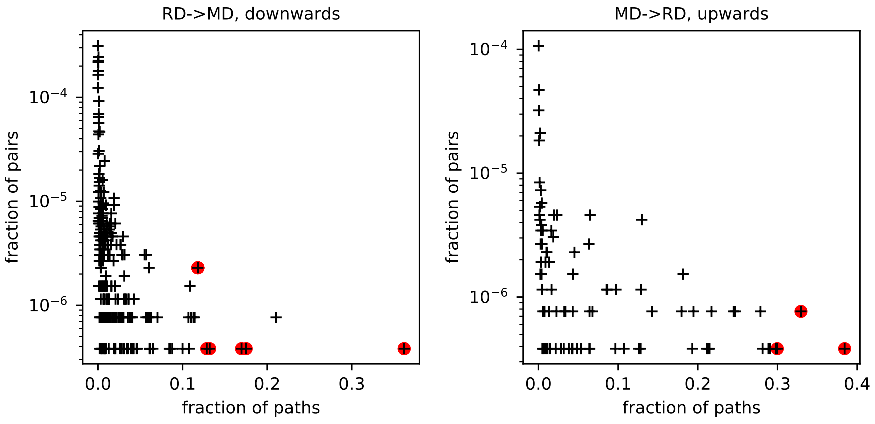

(c)

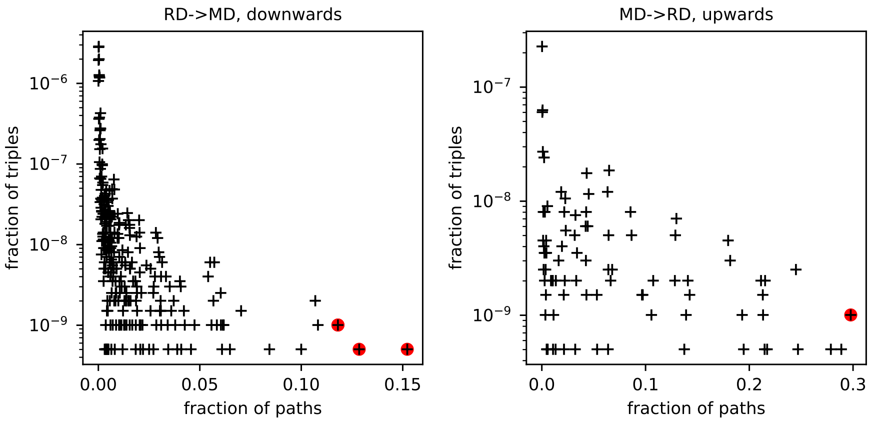

Fig E. Histograms of the fractions of (a) single vertices, (b) vertex pairs and (c) vertex triples in the PI passed by a certain fraction of the traversing paths. The bins containing the corresponding triplets from Table E (or pairs composed from the vertices of these triplets in (b) and the vertices themselves in (a)) are highlighted (hence the number of highlighted bins may be smaller than the number of entries in Table E). From Table E we obtain downwards: 4 triples, 11 pairs (excluding duplicates); 9 single vertices; upwards: 1 triples, 3 pairs; 3 single vertices.

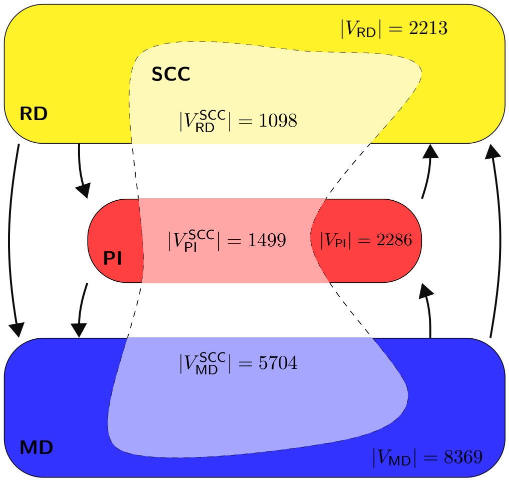

Fig F. Illustration of the largest strongly connected component (SCC) in our network model. The largest SCC is the only SCC to span all three domains.

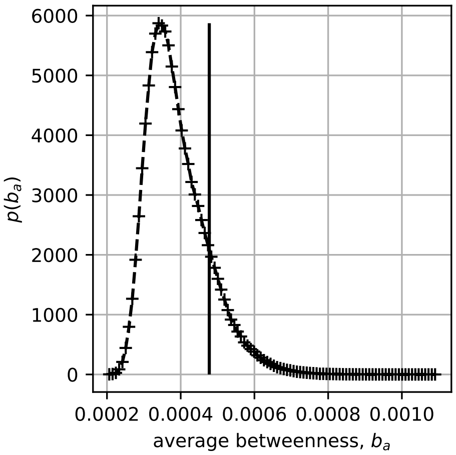

Fig G. Distribution of average betweenness centrality values of randomly chosen groups of vertices of the size of the protein interface. The distribution is based on 10^6 samples and consists of 100 equally spaced bins. The vertical line indicates the average value within the interface; about 0.84 of the distribution is below this value.

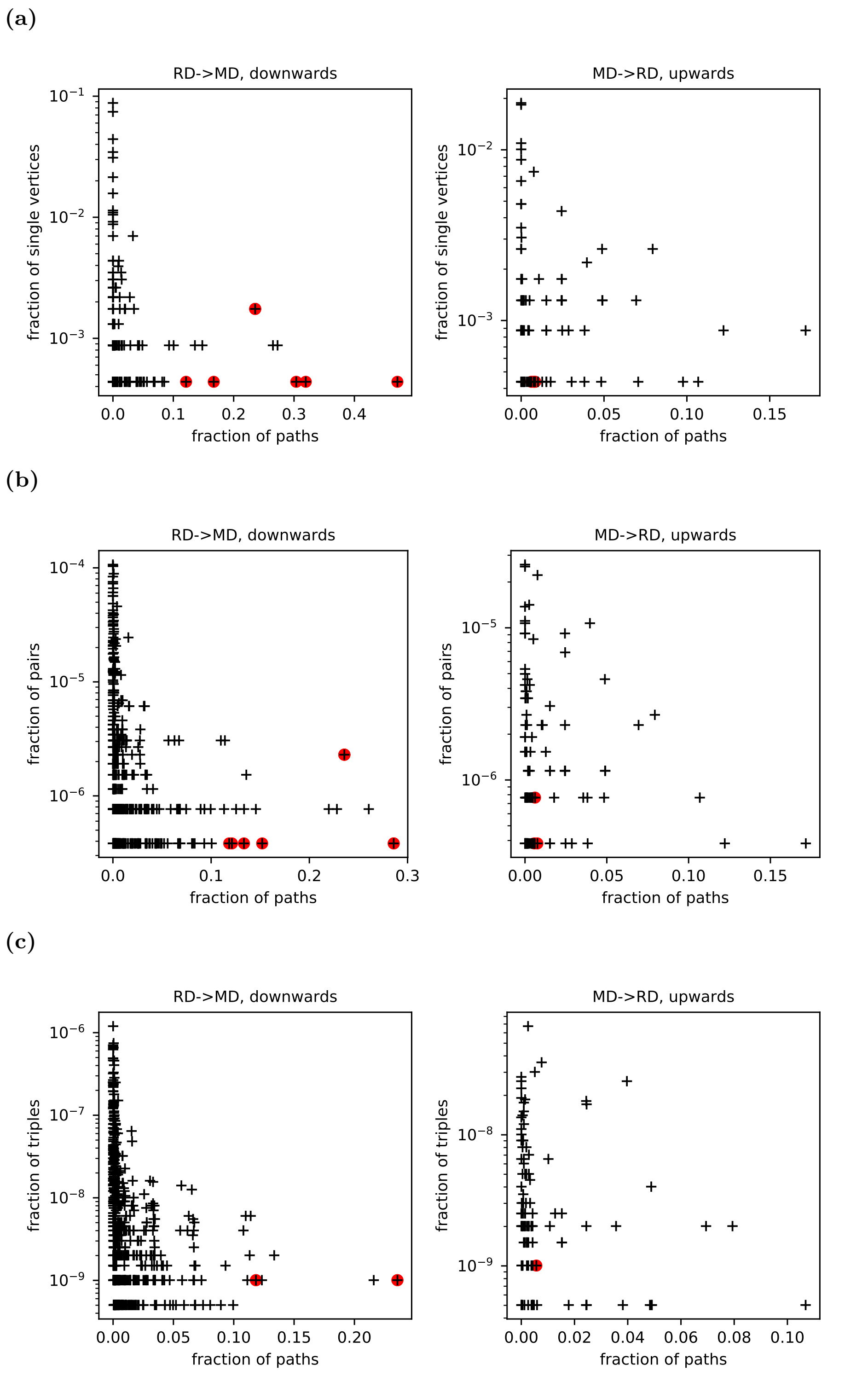

Fig H. Histograms of the fraction of feedback loops consisting of traversing paths passing through (a) single vertices, (b) pairs of vertices, (c) triples of vertices. As in Figure E, bins containing the corresponding triples from Table E are highlighted.

References

1. Covert MW, Knight EM, Reed JL, Herrgard MJ, Palsson B. Integrating high-throughput and computational data elucidates bacterial networks. Nature. 2004;429(6987):92–96. doi:10.1038/nature02456.

2. Feist AM, Henry CS, Reed JL, Krummenacker M, Joyce AR, Karp PD, et al. A genome-scale metabolic reconstruction for Escherichia coli K-12 MG1655 that accounts for 1260 ORFs and thermodynamic information. Molecular Systems Biology. 2007;3:121. doi:10.1038/msb4100155.

3. Gama-Castro S, Salgado H, Santos-Zavaleta A, Ledezma-Tejeida D, Muñiz-Rascado L, García-Sotelo JS, et al. RegulonDB version 9.0: high-level integration of gene regulation, coexpression, motif clustering and beyond. Nucleic Acids Research. 2015;44(D1):D133–D143. doi:10.1093/nar/gkv1156.

4. Klosik DF, Grimbs A, Bornholdt S, Hütt MT. The interdependent network of gene regulation and metabolism is robust where it needs to be. Nature Communications. 2017;8:534. doi:10.1038/s41467-017-00587-4.
